# JACQLQ subjective symptom questionnaire score and clinical test results for patients with allergic conjunctival disease

**DOI:** 10.1038/s41598-024-67117-3

**Published:** 2024-07-14

**Authors:** Yasuo Yamana, Satoshi Yamana, Eiichi Uchio

**Affiliations:** 1 Yamana Eye Clinic, Fukuoka, Japan; 2 Department of Ophthalmology, Graduate School of Medical Sciences, Kyushu University, Fukuoka, Japan; 3 Department of Ophthalmology, Faculty of Medicine, Fukuoka University, Fukuoka, Japan

**Keywords:** Allergic conjunctival disease, Tear total IgE, Serum-specific IgE, Serum total IgE, Quality of Life, Immunology, Medical research

## Abstract

We investigated the relationship between subjective symptoms and objective findings in patients with allergic conjunctival diseases (ACD) and test results for tear total IgE (t-tIgE), conjunctival eosinophils (c-Eo), serum total IgE (s-tIgE), serum-antigen specific IgE (s-sIgE), and serum eosinophils (s-Eo). Subjective symptoms and objective findings of patients with ACD were evaluated using Japanese Allergic Conjunctival Disease Quality of Life Questionnaire (JACQLQ), which described disability score and emotional score written by patient and clinical findings score written by ophthalmologist. We investigated the relationship between questionnaire scores and laboratory data for t-tIgE, c-Eo, s-tIgE, s-sIgE, and s-Eo. Scores of impediments to life and of moods were highest in vernal keratoconjunctivitis among ACD. Cases with positive pollen-sIgE showed significantly more nasal symptom score than those with negative pollen-sIgE (P < 0.05). Cases with positive t-tIgE or c-Eo showed significantly more objective symptoms’ JACQLQ score than those with negative t-tIgE or c-Eo (P < 0.05), respectively. Cases positive for house dust/mite-sIgE, showed significantly more objective symptoms’ JACQLQ score than those without for house dust/mite-sIgE (P < 0.05). These results indicate that ACD could be analyzed more accurately by the combination of JACQLQ and laboratory data.

## Introduction

Allergic diseases are inflammatory diseases of conjunctiva accompanied by conjunctival hyperemia and itching^1,2^. In recent years, allergic conjunctival diseases are thought to be increasing^2–5^. Not only IgE-related type I allergy but also air pollutants such as Asian dust, particulate matter 2.5 (PM2.5) and diesel exhaust gas are responsible for the increase in allergic conjunctival diseases^6–12^.

Allergic rhinitis not mediated by IgE has been reported^13,14^ in otolaryngological field.

Allergic conjunctival diseases not related to IgE has been reported^15^ and possible relation with group 2 innate lymphoid cells (ILC2s) have been proposed in some kind of allergic conjunctivitis^9^. The etiology and pathogenesis of allergic conjunctival disease are complex^9,16,17^.

Allergic conjunctival diseases (ACD) cause various obstacles in daily life and social life. However, there are few reports that have examined in detail the relationship between laboratory data (tear total IgE (t-tIgE)^17–19^, conjunctival eosinophils (c-Eo)^17–19^, serum-specific IgE (s-sIgE), serum total IgE (s-tIgE)) and subjective and clinical symptoms.

In this study, the subjective symptoms and objective findings of patients with ACD were evaluated using the Japanese Allergic Conjunctival Disease Quality of Life Questionnaire (JACQLQ), which described disability score and emotional score written by patient and clinical findings score written by ophthalmologist. A score for clinical findings was used in this study. We investigated the relationship between questionnaire scores and laboratory data for t-tIgE, c-Eo, s-tIgE, s-sIgE, and serum eosinophils (s-Eo). We also examined the relationship between clinical symptoms and test results.

## Methods

### Study design and participants

This study followed the ethical principles of the Declaration of Helsinki and was approved by the Ethics Committee of Japan Clinical Society of Diabetes (No. 22/10//2019–6). The study has been registered as the retrospective observational study in the UMIN Clinical Trials Registry (UMIN Trial ID: UMIN000041978). Informed consent was obtained from all individual participants included in this study^19^.

The subjects were 110 patients with ACD who visited Yamana Eye Clinic between 2019 and 2021, and consented to the examination. ACD was diagnosed from clinical ocular symptoms of ACD and if those were positive for c-Eo, definitive diagnosis of ACD was done^20^. Classification of ACDs was based on the guideline^20^. Ages ranged from 7 to 86 years (mean 55.5 years), with a male to female ratio of 1: 3.

### Measurement of allergen testing

Table 1 shows the questionnaire about subjective symptoms and disability in the JACQLQ questionnaire. The eye symptom score, nasal symptom score, disability score, and mood score were provided by patient and the clinical objective findings shown in Table 2 were provided by physician.

**Table 1:** Japanese allergic conjunctival disease quality of life questionnaire.

Category I. Subjective symptoms (scale 0 to 4)	
Symptom 01	Itchy eyes
Symptom 02	Foreign body sensation
Symptom 03	Hyperemia
Symptom 04	Watery eyes
Symptom 05	Eye discharge
Symptom 06	Runny nose
Symptom 07	Sneezing
Symptom 08	Nasal congestion
Symptom 09	Itchy nose
Category II. Quality of life (scale 0 to 4)	
QOL 01	Reduced productivity at work/home
QOL 02	Poor mental concentration
QOL 03	Reduced thinking power
QOL 04	Impaired reading of book/newspaper
QOL 05	Reduced memory loss
QOL 06	Limitation of outdoor life
QOL 07	Limitation of going out
QOL 08	Hesitation visiting friend or relatives
QOL 09	Reduced contact with friends or others by telephone or conversation
QOL 10	Not an easy person to be around
QOL 11	Impaired sleep
QOL 12	Tiredness
QOL 13	Fatigue
QOL 14	Frustration
QOL 15	Irritability
QOL 16	Depression
QOL 17	Unhappiness
Category III. Overall QOL scale (facial imaging scale)	

Each symptom and QOL were scaled 0 to 4 points, and 0 points for no symptom to 4 points for the most severe symptoms.

**Table 2:** The check lists to clinical objective findings of eye symptoms by physician.

Palpebral conjunctiva	Hyperemia, edema, follicle, papillae, giant papillae
Bulbar conjunctiva	Hyperemia, chemosis
Corneal limbus	Limbal edema, Trantas’ dots
Cornea	Epithelial lesions

Objective clinical findings were recorded and t-tIgE levels were measured with Allerwatch® Tear IgE (Wakamoto Pharmaceutical Co., Ltd., Tokyo, Japan) (AW) kit^18^, using immunochromatography^20,21^.

Antibody levels against 39 allergens (including eight allergens measured with ImmunoCAP Rapid) were assessed with the View Allergy 39® (Thermo Fisher Diagnostics K.K., Tokyo, Japan) (View39) kit, using a chemiluminescent enzyme immunoassay^22^. Judgement criteria of View 39 is following; Class 1 is false positive and ≥ 0.27 index; class 2 to 6 is positive and class 2 is ≥ 0.50 index; class 3 is ≥ 1.80 index; class 4 is ≥ 7.05 index; class 5 is 17.35 index; and class 6 is 29.31 index. The t-sIgE level was measured using FEIA: IgE-RIST (radio-immunosorbent test). The standard value of t-sIgE was < 170 IU/mL, and is the reference range that includes 95% of healthy people.^19^.

Conjunctival cytology using spatula was carried out on the palpebral conjunctiva of patients; the specimens were examined for the presence of eosinophils using Hansel staining and an optical microscope (Olympus Corporation, Tokyo, Japan).^17^.

We investigated whether there is a relationship between objective clinical findings and test results, t-tIgE, s-sIgE, c-Eo, s-Eo and s-tIgE. Table 3 shows the patients above the standard value for the allergic examination.

**Table 3:** Number of cases above the standard values for allergic examination.

Tear total IgE	Positive; 67 cases (60.9%)	Negative; 43 cases (39.1%)
Conjunctiva eosinophils	Positive; 19 cases (17.3%)	Negative; 89 cases (80.9%)
Serum total IgE	≥ 170 IU/mL; 36 cases (32.7%)	< 170 IU/mL; 74 cases (67.3%)
Serum specific IgE	Positive; 81 cases (73.6%)	Negative; 29 cases (26.4%)
Serum eosinophils	≥ 500/μL; 7 cases (6.4%)	< 500/μL; 103 cases (93.6%)

The JACQLQ comprises the following three domains: Domain I with 9 items on ocular or nasal symptoms; Domain II with 17 items on daily activity and psychological well-being; and Domain III with 5 items on overall condition scored by facial expression. Patients self-rated each symptom on a 5-point scale according to the severity, ranging from “none” (0 point) to “severe” (4 points).

The subjective symptom score was classified into 9 items for eye symptoms and nasal symptoms, and each item was classified into 5 points from 0 point for no symptoms to 4 points for the most severe symptoms (Table1). A total of 0 to 68 points were recorded for the 17 items of disability and mood.

The JACQLQ, including ocular symptom score, nasal symptom score, disability score and mood score was investigated.

Clinical evaluation of ocular findings was carried out according to the ocular clinical grading system reported previously^23^. Among ten objective ocular clinical findings of conjunctival, limbal, and corneal lesions, three findings, conjunctival papillae, conjunctival giant papillae and corneal epithelial lesions, were each graded on a 4-point scale (0 = none, 1 = mild, 2 = moderate, and 3 = severe; left and right eyes separately in each case). The total symptom score for both eyes’ ranged from 0 to a maximum of 60 points.

### Statistical analysis

A two-sided t-test was performed at a significance level of 5% to determine whether there was a significant difference between the patient’s questionnaire about subjective symptoms, the physician’s objective findings, and the mean of each test results. Wilcoxon’s rank sum test was also used in some analysis. Spearman’s rank correlation coefficient was calculated to evaluate the correlation between subjective and objective JACQLQ scores. All statistical analyses were carried out using R software (V4.1.2, www.r-project.org).

## Results

Among 110 cases of clinically diagnosed allergic conjunctivitis, 23.9% of them those with positive t-tIgE but negative s-tIgE and s-sIgE were considered as clinically diagnosed LACs, and of which 3 had positive c-Eo and they were diagnoses as definitive LAC (Table 4). Vernal keratoconjunctivitis (VKC) had mean scores of ocular symptoms 10 points, nasal symptoms 7.0 points, impediments to life 13.5 points, and moods 9.5 points, respectively (Table 5).

**Table 4:** Cases with clinically diagnosed allergic conjunctival diseases.

	Tear total IgE positive: 67cases	Tear total IgE negative: 43cases
Serum specific IgE positive: 81cases	51 cases (76.1%) *c-Eo positive of 15 cases	30 cases (69.8%) *c-Eo positive of 1 case
Serum specific IgE negative: 29cases	16 cases (23.9%) *c-Eo positive of 3 cases	13 cases (30.2%)

*c-Eo* conjunctival eosinophils.

**Table 5:** The comparison of mean JACQLQ ver.1 score between this study and Fukagawa’s study in 2012 (Fukagawa et al. 2012).

	Fukagawa’s multi-center study (2012): 648cases		Yamana Eye Clinice (2019–2021): 110cases	
	Score of eye symptoms	Score of nasal symptoms	Score of eye symptoms	Score of nasal symptoms
SAC	5.6 points	3.6 points	5.0 points	4.6 points
PAC	5.6 points	3.6 points	5.2 points	2.6 points
VKC	5.0 points	4.2 points	10.0 points	7.0 points
SAC + PAC			5.4 points	5.0 points
Total	4.8 points	3.1 points	5.2 points	3.8 points

	Fukagawa’s multi-center study (2012): 648cases		Yamana Eye Clinice (2019–2021): 110cases	
	Score of impediments of life	Score of moods	Score of impediments of life	Score of moods
SAC	5.4 points	3.9 points	5.4 points	2.7 points
PAC	5.2 points	3.9 points	4.7 points	3.1 points
VKC	7.0 points	3.7 points	13.5 points	9.5 points
SAC + PAC			8.3 points	4.1 points
Total	4.7 points	3.8 points	6.0 points	3.3 points

*SAC* seasonal allergic conjunctivitis, *PAC* perennial allergic conjunctivitis, *VKC*, vernal keratoconjunctivitis.

**Figure 1:**
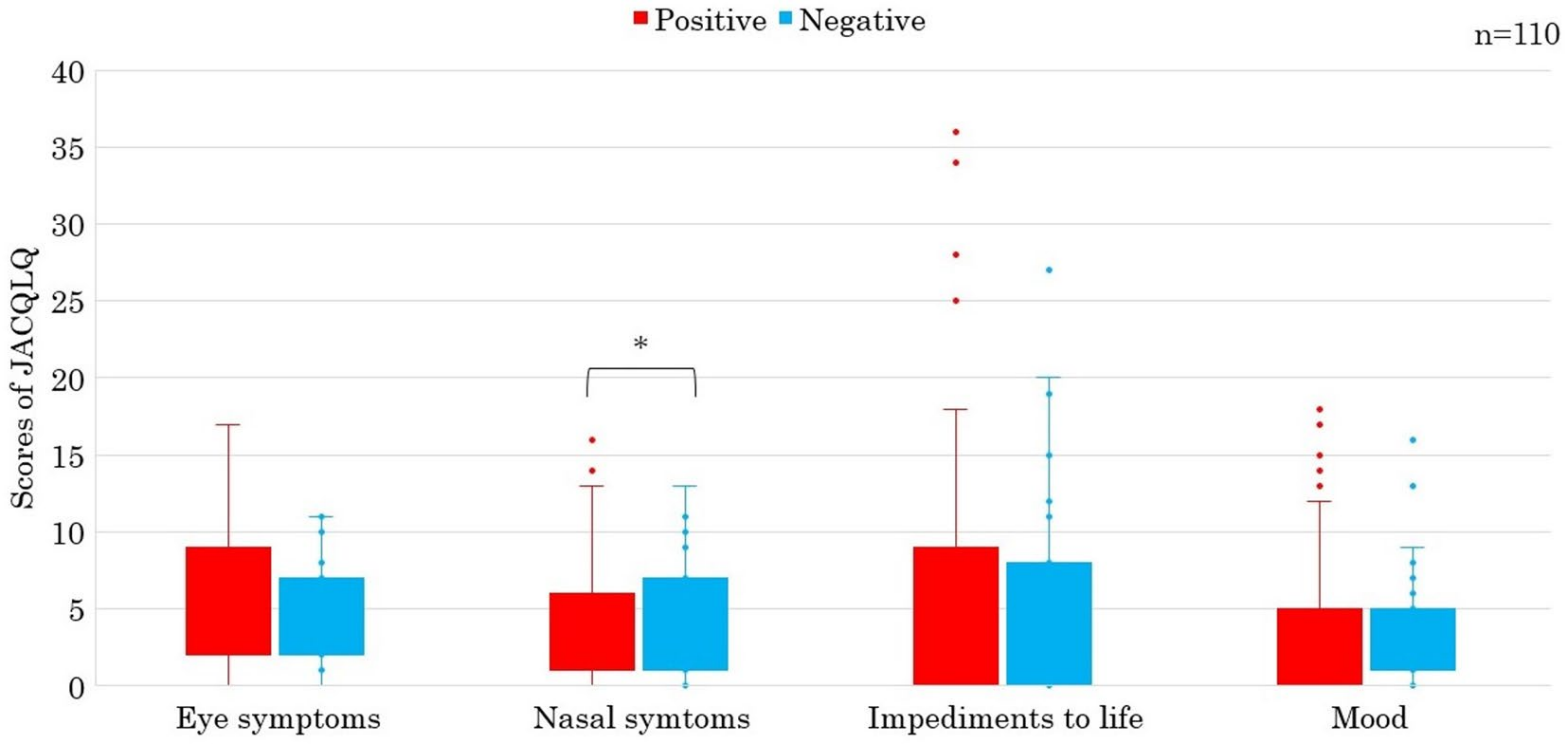
Comparison of subjective symptoms and quality of life’s score by JACQLQ ver1 between cases positive or negative for serum-specific IgE, * p < 0.05. Serum-specific IgE-negative patients had significantly higher Nasal symptoms’ scores (*p* < *0.05*).

**Figure 2:**
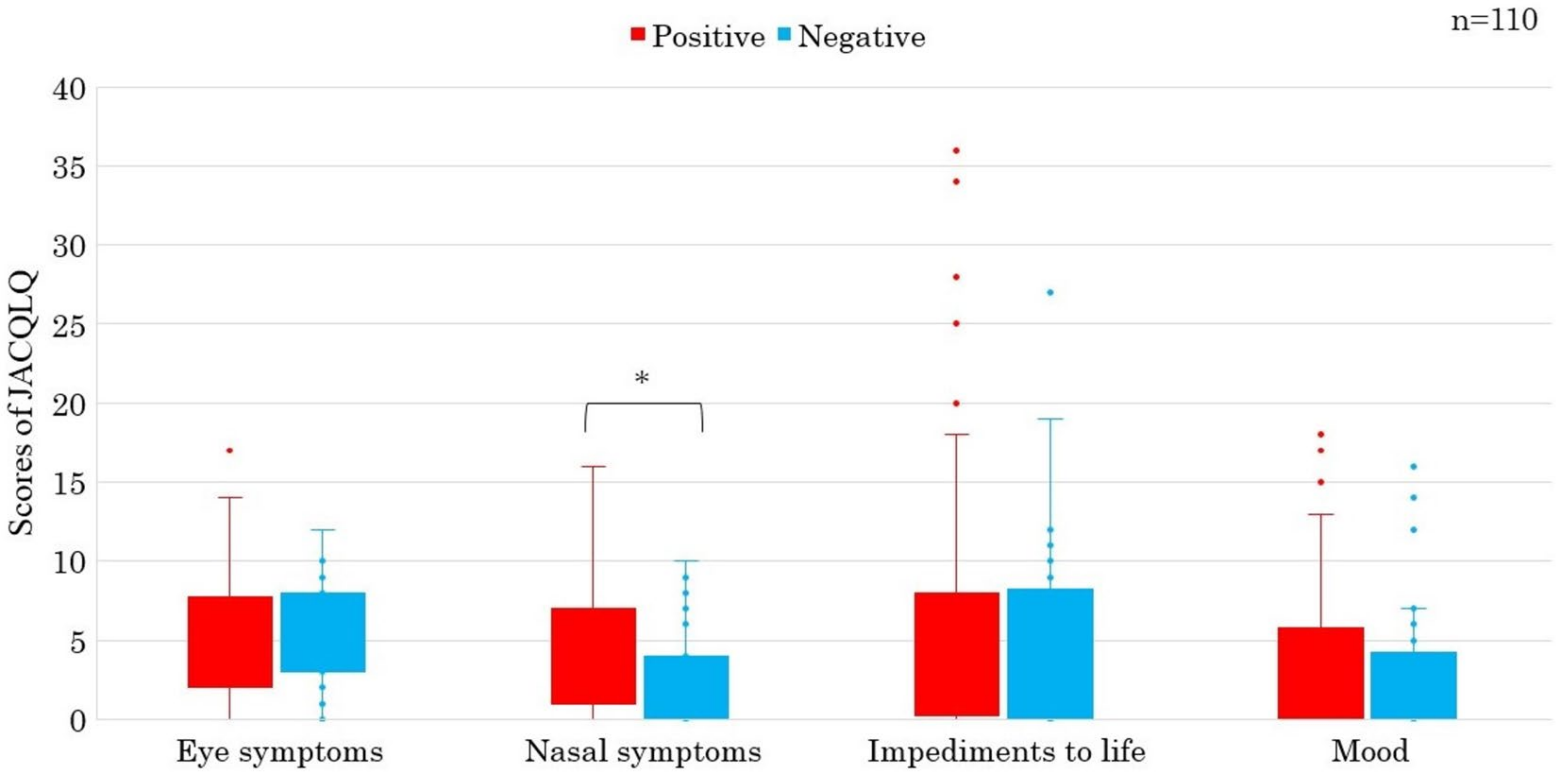
Comparison of subjective symptoms and quality of life’s score by JACQLQ ver1 between cases positive or negative for pollen-specific IgE, * p < 0.05. Pollen-specific IgE-positive patients had significantly higher nasal symptoms’ scores (*p* < *0.05*).

**Figure 3:**
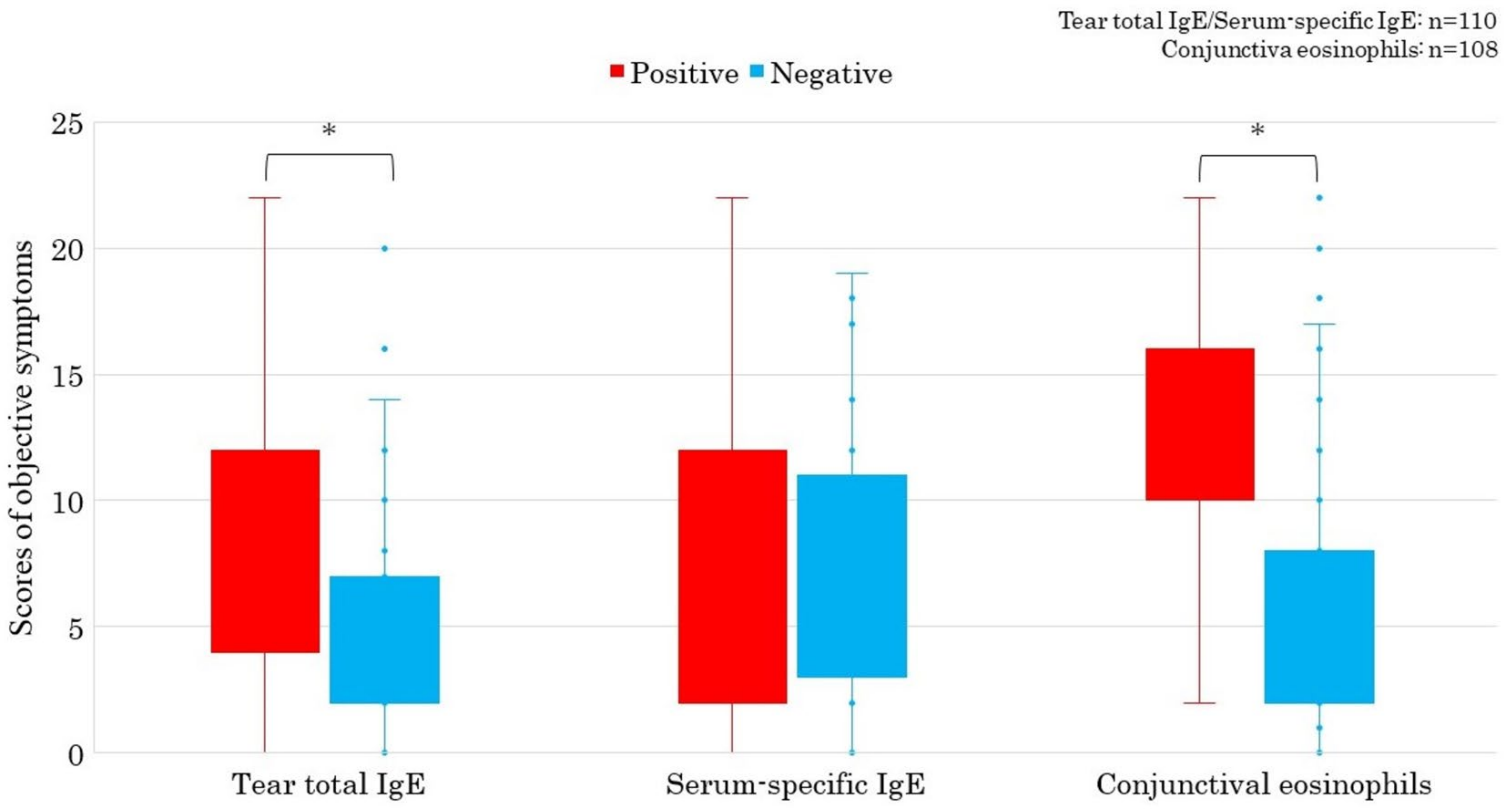
Comparison of objective symptoms’ scores by JACQLQ ver1 between cases positive or negative for tear total IgE, serum specific IgE and conjunctival eosinophils, * p < 0.05. Tear total IgE-positive and Conjunctival eosinophils-positive patients had significantly higher objective symptoms’ scores (*p* < *0.05*).

**Figure 4:**
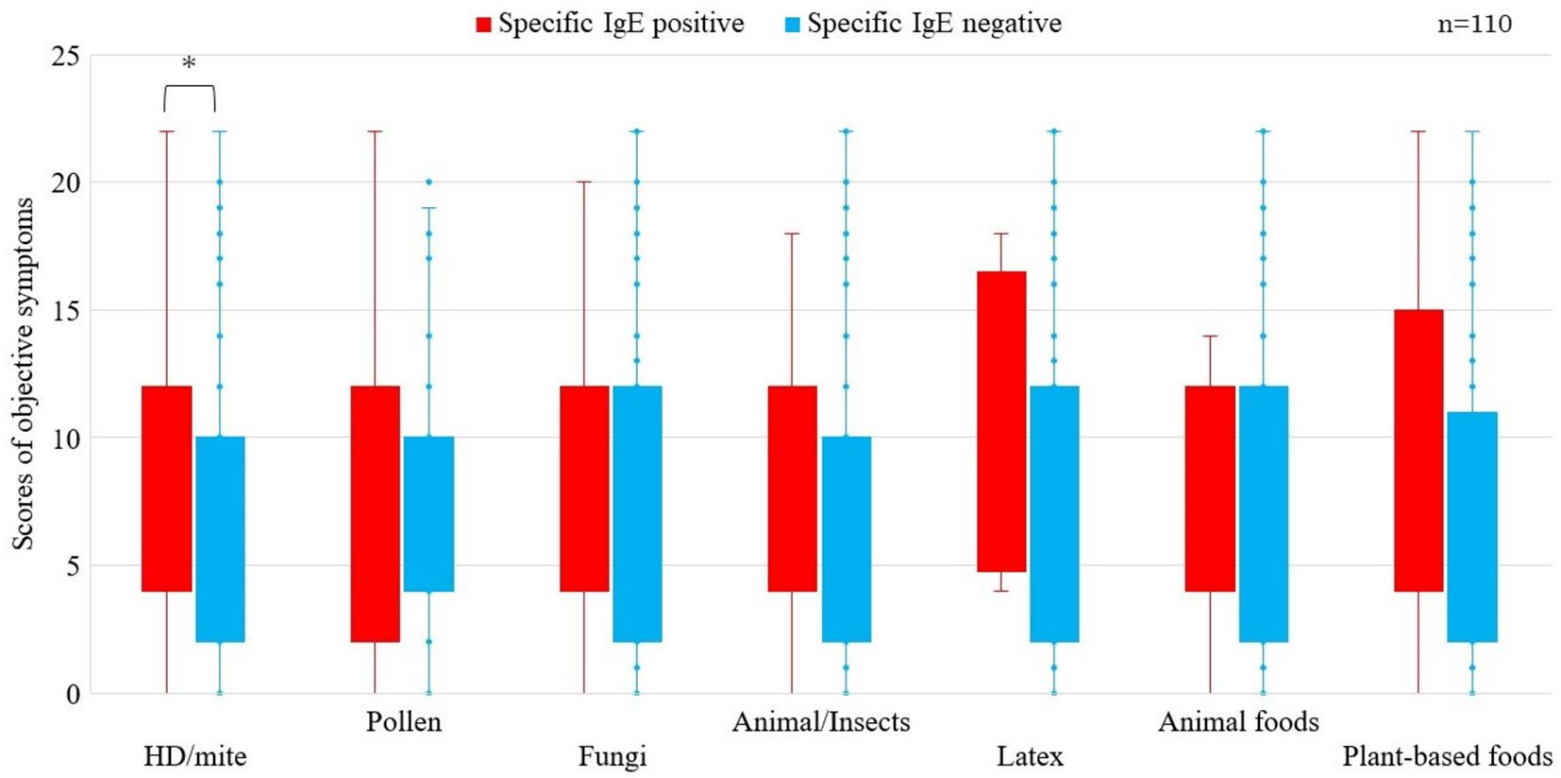
Objective symptoms score by JACQLQ ver1 between cases positive or negative for antigen specific IgE antibody, * p < 0.05. Serum-specific IgE-positive means that the antibodies were greater than the standard values in View39 test, and serum-specific IgE-negative means that those were blow the standard value. House dust/mite-specific IgE-positive patients had significantly higher objective symptoms’ scores (*p* < *0.05*). Significant difference was observed between cases with and without animal-insects or plant-based foods-specific IgE by Wilcoxon rank sum test (P < 0.05). HD, house dust.

**Figure 5:**
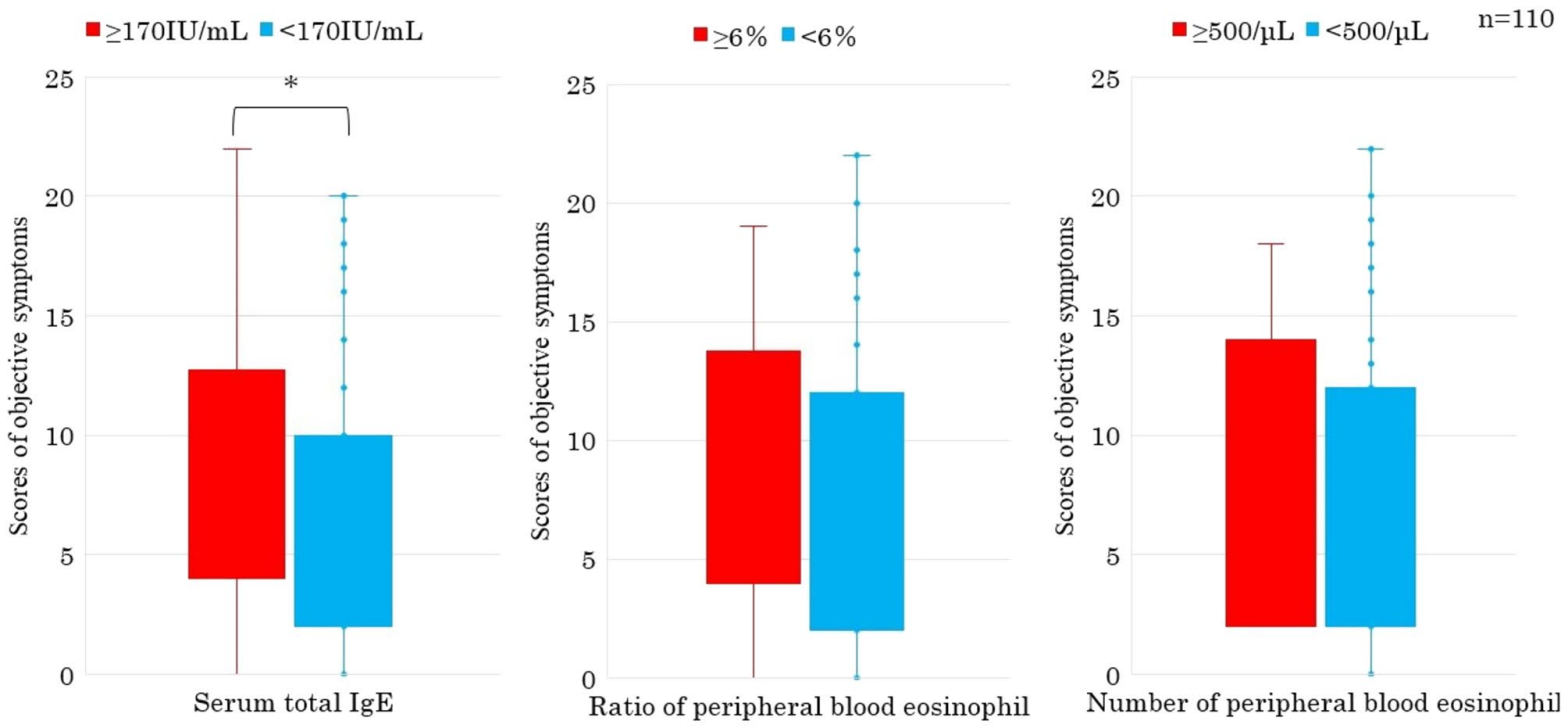
Comparison of objective symptoms by JACQLQ ver1 between high and low level groups of serum total IgE, ratio and number of peripheral blood eosinophils, * p < 0.05. Objective symptoms’ scores were significantly higher in patients with serum total IgE greater than 170 IU/mL (*p* < *0.05*).

Cases with negative s-sIgE showed significantly more nasal symptom score than those with positive s-sIgE (P < 0.05) (Fig. 1). Cases with positive pollen-sIgE showed also significantly more nasal symptom score than those with negative pollen-sIgE (P < 0.05) (Fig. 2). Cases with positive t-tIgE or c-Eo showed significantly more objective symptoms’ JACQLQ score than those with negative t-tIgE or c-Eo, respectively (P < 0.05). Significant difference was not observed in the score between cases of positive or negative for s-sIgE. (Fig. 3). Cases with positive t-tIgE or c-Eo showed significantly more objective symptoms’ JACQLQ score than those with negative t-tIgE or c-Eo, respectively (P < 0.05) (Fig. 3). Cases positive for house dust/mite-sIgE, showed significantly more objective symptoms’ JACQLQ score than those without for house dust/mite-sIgE (P < 0.05). Significant difference was observed between cases with and without animal-insects or plant-based foods-sIgE by Wilcoxon rank sum test (P < 0.05), although no difference was found by unpaired t-test (Fig. 4). Objective symptoms by JACQLQ between high and low level groups of serum total IgE showed significant difference (P < 0.05), while significant difference was not observed in other laboratory test results (Fig. 5).

**Figure 6:**
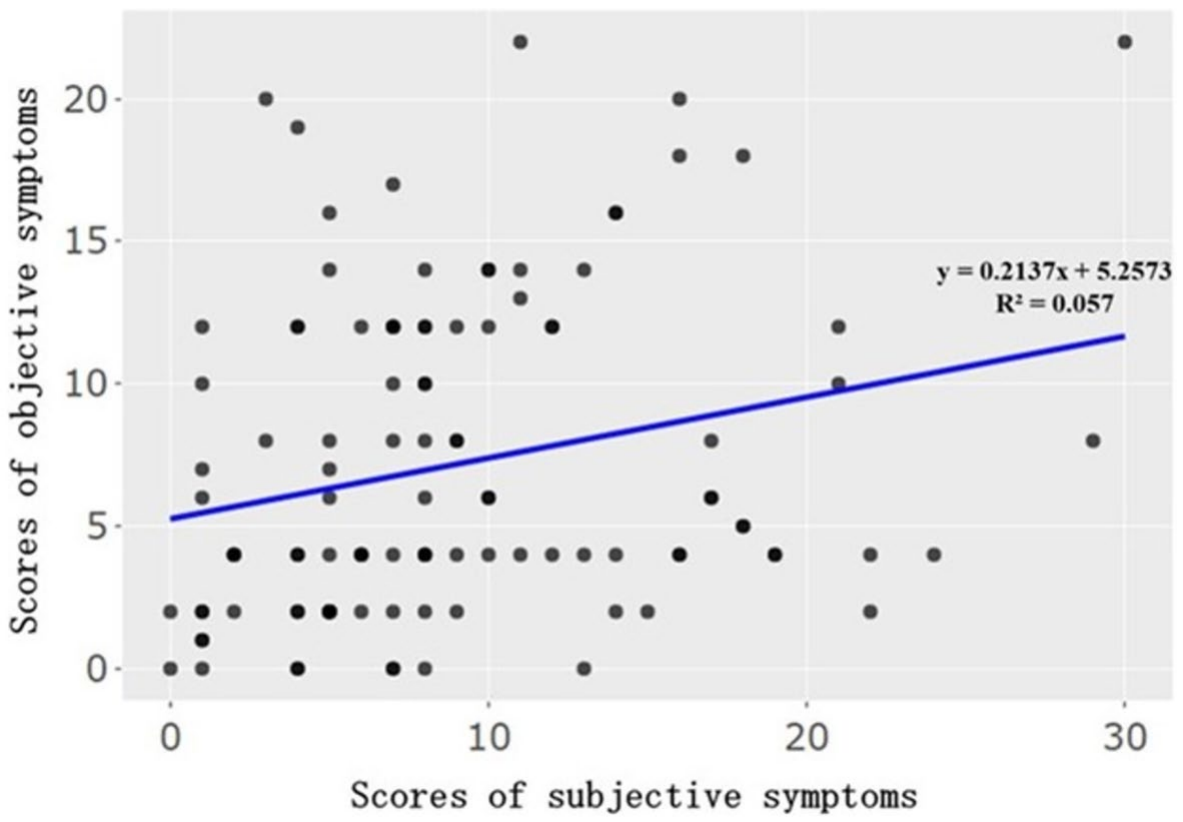
Correlation between subjective symptoms and objective symptoms. The slope of the linear regression equation was significant (p = 0.01).

Significant correlation was not found between subjective symptom score and objective symptom score (squared = 0.057), but the slope of the linear regression equation was significant (p = 0.01) (Fig. 6).

**Figure 7:**
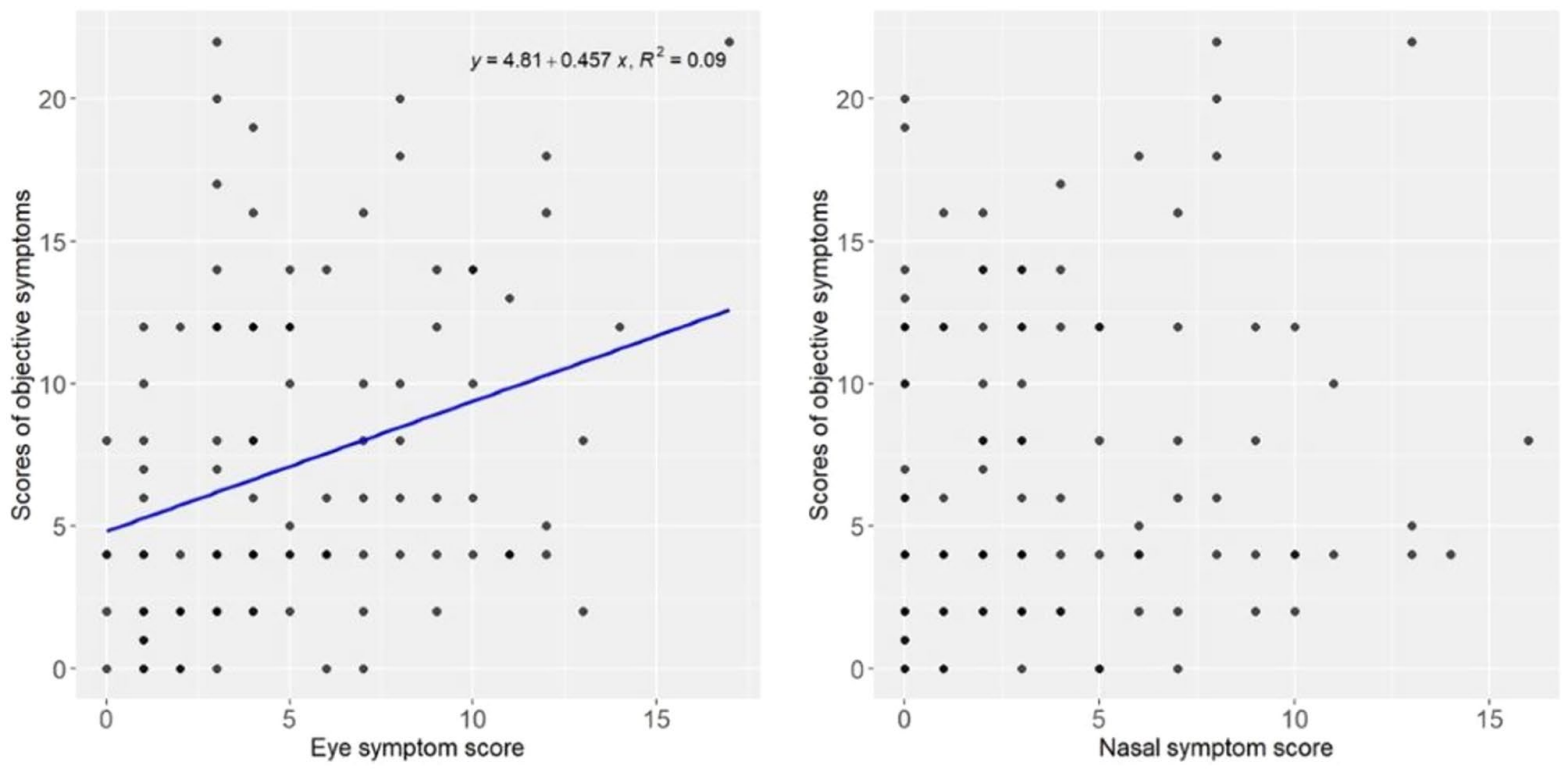
Correlation between objective symptoms and eye symptom total score (R2 = 0.09, p < 0.01) and objective symptoms and nasal symptom total score (R2 = 0.01, p = 0.29). Although the correlation between the total score of eye symptoms and the objective symptoms was not that strong, there was a somewhat weak correlation (R2 = 0.09, p < 0.01). There was no correlation between the subjective symptom score and the total score of nasal symptoms (R2 = 0.01, p = 0.29).

Although the correlation between the total score of eye symptoms and the objective symptoms was not that strong, there was a somewhat weak correlation (R2 = 0.09, p < 0.01). In terms of correlation, the subjective symptom score was somewhat strongly correlated with the eye symptom score. On the other hand, there was no correlation between the subjective symptom score and the total score of nasal symptoms (R2 = 0.01, p = 0.29). (Fig. 7).

## Discussion

It has been reported that allergen-specific IgE antibodies in tears were considered to be produced locally rather than the antibodies from serum^19,24,25^. We have reported that antigen sensitization likely to occur in conjunctiva in pollinosis, because positive rate of t-tIgE was significantly higher in patients with pollinosis than the positive rate of pollen-sIgE^19^.

In patients with ACD, various ocular symptoms are often accompanied by nasal symptoms^2^, asthma^5^, and ACD often interferes with patients’ daily life. Present results as also stated by Komiyama et al.^26^ that cases with positive pollen-sIgE showed significantly more nasal symptom score than those with negative pollen-sIgE (Fig. 2) might suggest nasal symptoms derived from allergic rhinitis have an important clinical or pathophysiological role in the QOL of patients with ACD. There is a correlation between seasonal onset groups of ACD and season specific antigens^24,25,27–29^. It was reported that treatment of ACD improved the quality of life by reducing t-tIgE and clinical symptoms^30,31^. However, few reports have examined the relationship between subjective symptoms and laboratory test results such as t-tIgE^17–19^, c-Eo^17–19^, s-sIgE and s-tIgE in details^30,32^.

Regarding allergy test results, although Inada et al.^21^ and Mimura et al.^33^ state that t-tIgE levels are similar to s-tIgE levels, in this study, the proportion of s-tIgE positivity was lower than the proportion of t-tIgE positivity. (Table 3) It has also been reported that there was no correlation between t-tIgE and s-tIgE, and this may be due to the difference in severity of allergic conjunctivitis patients at general clinics and hospitals^34^. However, as reported^33,35^ there is a relationship between positive t-tIgE and s-sIgE, this study also found a relationship between the two (Table 4).

We evaluated the subjective symptoms and quality of life scores (the JACQLQ eye symptom score, nasal symptom score, disability score, mood score, etc.)^36^ of patients with ACD at the time of clinic visit. Using JACQLQ questionnaire, Fukushima et al. reported that use of topical antihistamines may be an effective means of improving JACQLQ of patients with seasonal allergic conjunctivitis^37^, while the relationship between JACQLQ score and laboratory findings has not been mentioned.

The findings that cases with positive t-tIgE or c-Eo showed significantly more objective symptoms’ JACQLQ score than those with negative t-tIgE or c-Eo, respectively, indicates that the importance of local IgE and eosinophil in the development and deterioration of ACD which could be confirmed by subjective score system (Fig. 3).

In Table 5, the VKC ocular symptom score, nasal symptom score, impediments to life, and mood score were higher than those reported by Fukagawa et al.^38^. This may be because VKC is common in children, but the number of children in this study was small, or it may be due to regional characteristics^2^.

The Japanese guideline for ACD^20^ defines the presence of c-Eo as a definitive diagnosis of ACD if clinical symptoms were observed. C-Eo have been reported to be elevated in atopic conjunctivitis and VKC^39^. From the results that cases positive for house dust/mite-sIgE, showed significantly more objective symptoms’ JACQLQ score than those without for house dust/mite-sIgE house dust/mite, it might be suggested that house dust/mite plays an important role in the pathogenesis of ACD among various antigens studied, although this finding was not derived from tear sample but from serum sample (Fig. 4).

Significance in the slope of linear regression equation indicates that subjective symptom score and objective symptoms did not have direct correlation, but they have a kind of interaction, because objective symptoms score increase according to the increase of subjective symptoms (Fig. 6).

There was no correlation between the subjective symptom score and the total nasal symptom score (R2 = 0.01, P = 0.29). This suggests that most of the correlation in subjective symptoms is due to the eye symptom score. (Fig. 7).

## Conclusion

ACD could be analyzed more accurately by the combination of JACQLQ and laboratory data. Among various laboratory tests, ocular local data obtained from t-tIgE and e-Eo might be most important reflecting the pathogenesis of ACD. We believed that allergic conjunctivitis was caused by conjunctival sensitization.

## Data Availability

The datasets generated and analyzed in the current study are available from the corresponding author upon reasonable request.
